# Glucose Oscillations Can Activate an Endogenous Oscillator in Pancreatic Islets

**DOI:** 10.1371/journal.pcbi.1005143

**Published:** 2016-10-27

**Authors:** Joseph P. McKenna, Raghuram Dhumpa, Nikita Mukhitov, Michael G. Roper, Richard Bertram

**Affiliations:** 1 Department of Mathematics, Florida State University, Tallahassee, Florida, United States of America; 2 Department of Chemistry and Biochemistry, Florida State University, Tallahassee, Florida, United States of America; 3 Department of Chemistry and Biochemistry and Program in Molecular Biophysics, Florida State University, Tallahassee, Florida, United States of America; 4 Department of Mathematics and Programs in Neuroscience and Molecular Biophysics, Florida State University, Tallahassee, Florida, United States of America; Georgia Institute of Technology, UNITED STATES

## Abstract

Pancreatic islets manage elevations in blood glucose level by secreting insulin into the bloodstream in a pulsatile manner. Pulsatile insulin secretion is governed by islet oscillations such as bursting electrical activity and periodic Ca^2+^ entry in *β*-cells. In this report, we demonstrate that although islet oscillations are lost by fixing a glucose stimulus at a high concentration, they may be recovered by subsequently converting the glucose stimulus to a sinusoidal wave. We predict with mathematical modeling that the sinusoidal glucose signal’s ability to recover islet oscillations depends on its amplitude and period, and we confirm our predictions by conducting experiments with islets using a microfluidics platform. Our results suggest a mechanism whereby oscillatory blood glucose levels recruit non-oscillating islets to enhance pulsatile insulin output from the pancreas. Our results also provide support for the main hypothesis of the Dual Oscillator Model, that a glycolytic oscillator endogenous to islet *β*-cells drives pulsatile insulin secretion.

## Author Summary

A global shift throughout the last century toward excessive nutrient intake relative to energy expenditure has fueled a dramatic increase in the incidence of diabetes in humans. The epidemic is primarily of type 2 diabetes, a disease characterized by the inability of the body to effectively control blood glucose levels. Insulin plays a key role in regulating blood glucose levels by restraining endogenous glucose output from the liver and promoting blood glucose uptake by tissue throughout the body. It is secreted in pulses by islets of Langerhans, endocrine cell aggregates dispersed throughout the pancreas. Loss of insulin pulsatility is an early event in the development of type 2 diabetes. Here, we demonstrate, with a combined modeling and experimental approach, that the loss of pulsatile insulin release that results from elevated glucose may be recovered by an oscillatory glucose stimulus. Our results have potential implications for enhancing insulin pulsatility and therefore mitigating the development of type 2 diabetes.

## Introduction

Pancreatic islets manage elevations in blood glucose level, such as after meals, by secreting insulin into the bloodstream, which enables the liver, muscles, and adipose tissue to take up glucose. Insulin is secreted from islet *β*-cells in a pulsatile manner governed by oscillations in intracellular Ca^2+^ concentration, which are caused by bursting electrical activity on the membrane [1]. Pulsatile insulin secretion enhances the role of the liver in glucose homeostasis [2–4], and insulin pulsatility is impaired in patients with type 2 diabetes [5–7]. In healthy subjects, blood insulin levels are oscillatory with a period of approximately 5 min in both the hepatic portal vein proximal to the pancreas [8–10] and peripheral arteries [11, 12], indicating that the several hundred thousand islets in a rodent or human pancreas [13] synchronize to produce pulsatile insulin output to the portal vein.

In response to glucose, some islets exhibit fast Ca^2+^ oscillations with periods of less than 2 min, some exhibit slow Ca^2+^ oscillations with periods of greater than 2 min, and some exhibit compound Ca^2+^ oscillations that consist of episodes of fast oscillations interrupted with silent phases (see [14] and [15] for reviews). This variety of responses suggests that two oscillators cooperate in islets: a “slow oscillator” with period corresponding to that of slow Ca^2+^ oscillations or episodes of compound Ca^2+^ oscillations (> 2 min) and a “fast oscillator” with period corresponding to that of fast Ca^2+^ oscillations or intra-episode oscillations of compound Ca^2+^ oscillations (< 2 min). In this report, we focus on slow oscillations since, with a period similar to that of pulsatile insulin secretion [16], they are a potential target for enhancing pulsatility.

**Fig 1:**
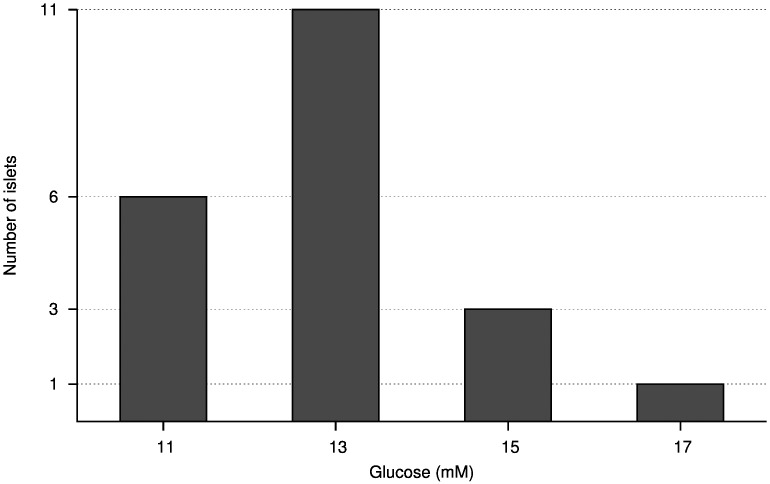
Heterogeneity of islet slow oscillators. The glucose concentration at which slow Ca^2+^ oscillations were lost as glucose was gradually increased. Out of 34 islets, 21 islets exhibited Ca^2+^ oscillations with a period > 2 min. Of those 21 islets, the concentration at which slow Ca^2+^ oscillations were lost varied from 11 to 17 mM (mean: 12.90 mM, standard deviation: 2.47 mM).

At low concentrations of glucose, the islet slow oscillator is inactive and electrical activity is absent, which results in no more than basal insulin secretion. When glucose is continuously increased, the slow oscillator activates at a low threshold (≈ 5 mM), which results in slow or compound Ca^2+^ oscillations and pulsatile insulin secretion. As glucose is increased further, the slow oscillator deactivates although electrical activity may persist, which results in elevated intracellular Ca^2+^ and constant insulin secretion [17]. We found that slow oscillators are heterogeneous across islets by recording the glucose level at which slow Ca^2+^ oscillations were lost as we gradually increased external glucose (Fig 1). Out of 34 islets tested, 21 islets exhibited oscillations with a period of 2 min or greater. Out of those 21 islets, the glucose level at which slow Ca^2+^ oscillations were lost varied from 11 to 17 mM, indicating that, *in vivo*, elevated blood glucose levels may trigger pulsatile insulin secretion from a sub-population of islets in the pancreas but continuous secretion from the remaining islets, which would attenuate insulin pulsatility.

In this report, we demonstrate that slow Ca^2+^ oscillations lost by fixing glucose at an elevated concentration may be recovered by subsequently oscillating glucose sinusoidally about that concentration with sufficiently large amplitude and period. Slow oscillations are often recovered after a delay or with a period that differs significantly from that of the oscillatory glucose signal, indicating that the glucose signal activates an endogenous oscillator. Complex patterns of islet entrainment by sinusoidal glucose, including a 1:2 ratio between the frequencies of Ca^2+^ and imposed glucose oscillations, have previously been reported [18].

We previously demonstrated that blood glucose level fluctuations [19] could reflect interactions between the pancreas and the liver [20]. The liver plays a negative feedback role important to managing blood glucose levels. It removes glucose from the bloodstream when blood insulin is elevated (which is caused by elevated blood glucose) and releases glucose to the bloodstream when blood insulin level is low (which is caused by low blood glucose) [21]. Therefore, a sub-population of oscillating islets in the pancreas delivering an oscillatory insulin signal to the liver could establish blood glucose level oscillations of a similar period that recruit non-oscillating islets to enhance insulin pulsatility. In this report, we demonstrate that a sub-population of slow-oscillating islets can recruit a sub-population of fast-oscillating islets when glucose delivery is put under the control of a model liver that supplies negative feedback to insulin secretion.

We use a mathematical model, the Dual Oscillator Model (DOM), to simulate experiments with islets and an *ex vivo* microfluidic platform to conduct experimental studies with islets. Our model simulations predict our experimental results and give further support to the main hypothesis of the DOM, that an endogenous glycolytic oscillator drives slow islet oscillations and pulsatile insulin secretion.

## Methods

### Chemicals and reagents

Potassium chloride (KCl), sodium chloride (NaCl), calcium chloride (CaCl_2_), magnesium chloride (MgCl_2_), tricine, and penicillin-streptomycin were purchased from Sigma-Aldrich (Saint Louis, MO, USA). Pluronic F-127 and fura-2-acetoxymethyl ester (fura-2-AM) were purchased from Life Technologies (Grand Island, NY, USA). Glucose (dextrose) was purchased from Thermo Fisher Scientific (Waltham, MA, USA). RPMI 1640 was purchased from Mediatech (Manassas, VA, USA). Gentamicin sulfate was purchased from Lonza (Basel, Switzerland). Poly(dimethylsiloxane) (PDMS) prepolymer (Sylgard 184) was purchased from Dow Corning (Midland, MI, USA). All solutions were made with Milli-Q (Millipore, Bedford, MA, USA) 18 MΩ cm deionized water.

### Isolation and culture of islets of Langerhans

The isolation of mouse islets of Langerhans was performed under guidelines approved by the Florida State University Animal Care and Use Committee (protocol 1235). Islets of Langerhans were isolated in a similar manner as described before [20]. After isolation, islets were incubated at 37°C, 5% CO_2_ in RPMI 1640 media containing 11 mM glucose, 10% calf serum, 100 units mL^−1^ penicillin, 100 *μ*g mL^−1^ streptomycin, and 10 *μ*g mL^−1^ gentamicin. Islets were used within 5 days after isolation.

### Microfluidic device

A microfluidic device was used to deliver different glucose concentrations to islets using the system described before [20]. Briefly, the device consisted of two inputs that were connected to 60 mL syringes that contained a balanced salt solution (BSS) composed of 2.4 mM CaCl_2_, 125 mM NaCl, 1.2 mM MgCl_2_, 5.9 mM KCl, and 25 mM tricine at pH 7.4 with either 3 or 20 mM glucose. The syringes were suspended above the microfluidic device where the elevated heights produced a hydrostatic pressure that controlled the flow rates of the reagents in the device. A stepper motor controlled by a LabVIEW (National Instruments, Austin, TX) program was used to control the height of the syringe containing 20 mM glucose. The height of the other syringe was coupled to this syringe using a fixed length belt and pulley system. In this way, the height of a single syringe was controlled while the other syringe moved in an equal, but opposite, direction maintaining a constant total flow rate in the device. The difference in the heights of the two syringes caused different flow rates of the two glucose solutions to enter a 70 mm mixing channel prior to delivery to a 0.8 mm diameter chamber that housed 4–10 islets. By changing the heights of the two syringes, the concentration of glucose delivered to the islets could be changed in ≈ 30 s. In most experiments, the glucose level is initially held fixed at 11 mM or greater, and then sinusoidal oscillations are applied. We hold at these relatively high levels since our goal is to examine behavior near the upper threshold for slow oscillations.

### Measurement of intracellular Ca^2+^

The microfluidic device was fixed on the stage of a Nikon Eclipse Ti inverted microscope. A Lambda XL (Sutter Instruments, Novato, CA) system containing a lamp and appropriate filters was used for excitation of fura-2 at 340 nm and 380 nm. Fluorescent images were acquired with a 150 ms exposure every 20 s using a CCD (Cascade, Photometrics, Tucson, AZ, USA). The imaging system was controlled by Nikon NIS Elements software (Nikon, Melville, NY, USA). The ratio of fluorescence intensity excited at 340 nm to that at 380 nm (FCO_340_/FCO_380_) for all islets was obtained from the NIS software and converted to intracellular Ca^2+^concentration using predetermined calibration values that were found by standard methods [20].

Loading of islets with fura-2 dye was performed by incubating islets in 2.5 *μ*M fura-2-AM and 0.02% Pluronic F-127 in RPMI for 40 min at 37°C and 5% CO_2_. Islets were then removed and placed in the microfluidic device where they were rinsed with BSS in 3 mM glucose for 5 min prior to the start of experiments.

### Mathematical modeling

**Fig 2:**
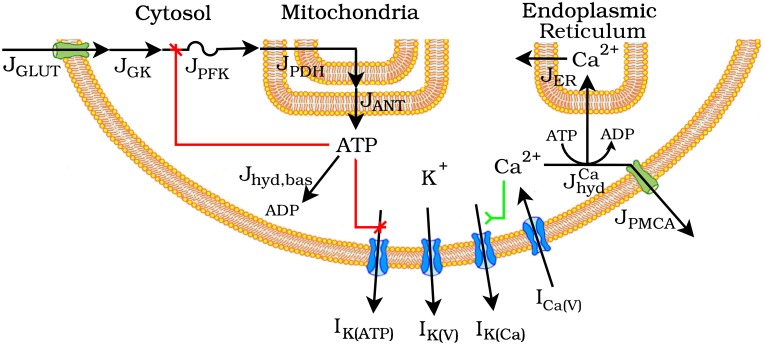
Schematic of the Dual Oscillator Model. J: flux; GLUT: glucose transporter, GK: glucokinase, PFK: phosphofructokinase, PDH: pyruvate dehydrogenase, ANT: adenine nucleotide translocator, ER: endoplasmic reticulum, hyd: hydolysis, PMCA: plasma membrane Ca^2+^ ATPase. I: current; Ca(V): voltage-activated Ca^2+^ K(V): voltage-activated K^+^, K(Ca): Ca^2+^-activated K^+^, K(ATP): ATP-sensitive K^+^.

We use the Dual Oscillator Model (DOM) [1, 14] to simulate islet *β*-cells. A model schematic is depicted in Fig 2. See S1 Model Equations for a full list of model equations and S1 Model Parameter Values for parameter values.

The central element of the DOM is a glycolytic subsystem, adapted from [22], that models the kinetics of the reaction catalyzed by the enzyme phosphofructokinase-1 (PFK1). Influx to the glycolytic subsystem is through glucose transporters (GLUT) that take up external glucose, *G*_*e*_, to intracellular glucose, *G*_*i*_. Efflux is through the mitochondrial pyruvate dehydrogenase (PDH) complex, which decarboxylates the final glycolytic product pyruvate to supply acetyl CoA to the citric acid cycle. The glycolytic model is a system of differential equations for the rates of concentration change of *G*_*i*_ and PFK1 substrate, fructose 6-phosphate (F6P), and product, fructose 1,6-bisphosphate (FBP):

$$\begin{array}{cc} dG_{i}/dt & =J_{\text{GLUT}}\left(G_{e},G_{i}\right)-J_{\text{GK}}\left(G_{i}\right)\\ d\text{F6P}/dt & \propto J_{\text{GK}}\left(G_{i}\right)-J_{\text{PFK}}\left(\text{F6P},\text{FBP},\text{ATP}\right)\\ d\text{FBP}/dt & \propto J_{\text{PFK}}\left(\text{F6P},\text{FBP},\text{ATP}\right)-J_{\text{PDH}}\left(\text{FBP}\right) \end{array}$$

derived by assuming the glucose 6-phosphate isomerase reaction upstream to PFK1 and the series of reactions downstream to PFK1 are at equilibrium [23, 24]. The glycolytic subsystem is coupled through ATP to a conductance-based model of electrical activity on the plasma membrane: a rise in glycolytic flux causes ATP production that reduces ATP-sensitive K^+^(K(ATP)) membrane ion channel conductance. The concentration of cytosolic ATP increases as adenine nucleotide translocators (ANT) export ATP from the mitochondrial matrix to the cytosol and decreases with hydrolysis (hyd):

$$\begin{array}{cc} d\text{ATP}/dt & =J_{\text{ANT}}\left(\text{FBP},\text{ATP},Ca_{c}\right)-J_{\text{hyd}}\left(\text{ATP},Ca_{c}\right). \end{array}$$

ATP hydrolysis powers pumping of cytosolic Ca^2+^, *Ca*_*c*_, across the endoplasmic reticulum (ER) and plasma membranes. The conductance-based model of membrane electrical activity, previously described in [25], includes voltage-activated Ca^2+^ (Ca(V)) and K^+^(K(V)) currents, Ca^2+^-activated K^+^current (K(Ca)), and K(ATP) current as well as Ca^2+^ flux across the ER and plasma membranes.

Model equations were integrated using the backward differentiation formulas implemented by the CVODE software package. The model can be downloaded as freeware from http://www.math.fsu.edu/~bertram/software/islet.

## Results

### Glycolytic oscillations in the Dual Oscillator Model

**Fig 3:**
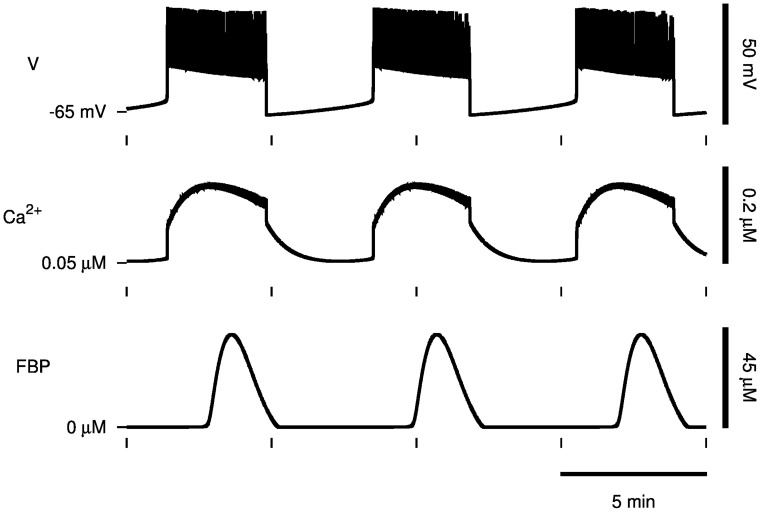
Slow oscillations in the Dual Oscillator Model. In the DOM, glycolytic oscillations such as in the concentration of PFK product, FBP, (bottom) lead to bursting oscillations in membrane potential (top) and oscillations in intracellular Ca^2+^ concentration (middle).

Our model, the Dual Oscillator Model (DOM), of islet *β*-cells contains a glycolytic subsystem that can produce oscillations with a period of approximately 5 min. Glycolytic oscillations in *β*-cells lead to oscillations in the intracellular ATP concentration which, by acting on ATP-sensitive K^+^ (K(ATP)) channels, cause bursting electrical activity on the membrane and oscillations in intracellular Ca^2+^ concentration (Fig 3).

**Fig 4:**
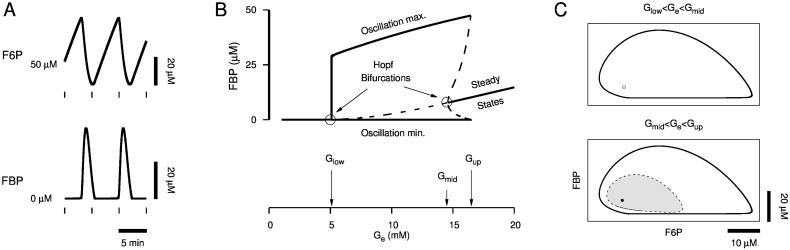
Glycolytic oscillations in the Dual Oscillator Model. (A) Concurrent sawtooth F6P oscillations and FBP pulses produced by the glycolytic subsystem of the DOM. (B) Bifurcation diagram of the glycolytic subsystem. Sustained oscillations do not exist for low (*G*_*e*_ < *G*_low_) or high (*G*_*e*_ > *G*_up_) glucose, but can be triggered for an intermediate range (*G*_low_ < *G*_*e*_ < *G*_up_). (C) Phase planes of the glycolytic subsystem within monostable (top) and bistable (bottom) glucose ranges. Initial states in the stable limit cycle (top and bottom, solid black curve) domain of attraction (top and bottom, white) trigger sustained oscillations. Initial states in the stable steady state (bottom, filled dot) domain of attraction (bottom, shaded gray) lead to transient, decaying oscillations. An unstable steady state (top, open dot) gains stability and gives rise to an unstable limit cycle (bottom, dashed curve) via a Hopf bifurcation as *G*_*e*_ increases past *G*_mid_.

Glycolytic oscillations are mediated by the autocatalytic activation of the enzyme PFK1 by its product, FBP. A rise in PFK1 substrate, F6P, from glucose consumption leads to FBP production, which positively feeds back onto PFK1 to increase its catalytic activity. Increased PFK1 catalytic activity eventually depletes F6P, leading to a crash in the FBP level and a recovery in F6P, completing one cycle of the oscillation. Ultimately, this mechanism coordinates sawtooth oscillations of F6P with pulses of FBP (Fig 4A). Which glucose levels and which initial states of the glycolytic subsystem produce sustained oscillations? Fig 4B depicts a bifurcation diagram for the asymptotic value of FBP with external glucose, *G*_*e*_, as a bifurcation parameter. The minimum and maximum values of FBP for periodic states of the glycolytic subsystem are represented by curves below and above the steady state curve, respectively, for *G*_low_ < *G*_*e*_ < *G*_up_. Stable states are represented by solid curves, whereas unstable states are represented by dashed curves.

The glycolytic subsystem is at steady state with FBP constant for low (*G*_*e*_ < *G*_low_) or high (*G*_*e*_ > *G*_up_) glucose. It becomes oscillatory by, say, continuously increasing *G*_*e*_ from a low concentration to a concentration between *G*_low_ and *G*_mid_ or by continuously decreasing *G*_*e*_ from a high concentration to a concentration between *G*_low_ and *G*_mid_. In either scenario, the steady state loses stability via a Hopf bifurcation, at *G*_*e*_ = *G*_low_ or *G*_*e*_ = *G*_mid_, and remains unstable for *G*_low_ < *G*_*e*_ < *G*_mid_, represented as the dashed portion of the steady state curve in Fig 4B.

The glycolytic subsystem can produce oscillations for an intermediate range of glucose (*G*_low_ < *G*_*e*_ < *G*_up_) that extends above *G*_mid_ to a saddle node of periodics bifurcation at *G*_*e*_ = *G*_up_. For *G*_low_ < *G*_*e*_ < *G*_mid_, the system is monostable with co-existing unstable steady state and stable limit cycle (Fig 4C, top), yet as *G*_*e*_ increases above *G*_mid_ so that *G*_mid_ < *G*_*e*_ < *G*_up_, the system becomes bistable as the steady state gains stability and gives rise to an unstable limit cycle via the subcritical Hopf bifurcation at *G*_*e*_ = *G*_mid_ (Fig 4C, bottom).

All initial states in the stable limit cycle’s domain of attraction trigger sustained oscillations. In the oscillatory, monostable range (*G*_low_ < *G*_*e*_ < *G*_mid_), the domain of attraction is the whole state space except the unstable steady state, and in the bistable range (*G*_mid_ < *G*_*e*_ < *G*_up_), it is the set of initial states outside the unstable limit cycle (depicted as a dashed line in Fig 4C, bottom). In the bistable range, initial states in the stable steady state’s domain of attraction (shaded gray in Fig 4C, bottom) produce transient oscillations that decay to the stable steady state. The existence of a subcritical Hopf bifurcation at *G*_*e*_ = *G*_mid_ is key to recovering slow Ca^2+^ oscillations with a sinusoidal glucose stimulus as we explain in the following sections.

The number and type of bifurcations of the glycolytic subsystem are robust with respect to increases in the maximal rate of GLUT transporter (*V*_GLUT_) or maximal rate of glucokinase (*V*_GK_). All of *G*_low_, *G*_mid_, and *G*_up_ are decreasing functions of *V*_GLUT_ and *V*_GK_. For example, doubling the value of *V*_GLUT_ = 0.01 mM/ms used throughout this report decreases *G*_mid_ by 25.2% to 10.9 mM. Similarly, doubling the value of *V*_GK_ = 0.01 mM/ms used throughout this report decreases *G*_mid_ by 16.2% to 12.2 mM.

### Sinusoidal variation in glucose can activate the glycolytic oscillator

In the glycolytic subsystem of the DOM, sustained oscillations are produced over an intermediate glucose range (*G*_low_ < *G*_*e*_ < *G*_mid_), but they may be lost by increasing glucose to elevated concentrations (*G*_*e*_ > *G*_mid_) (see Fig 4B). We found that sustained oscillations can be recovered by subsequently varying *G*_*e*_ sinusoidally with sufficiently large amplitude and period.

**Fig 5:**
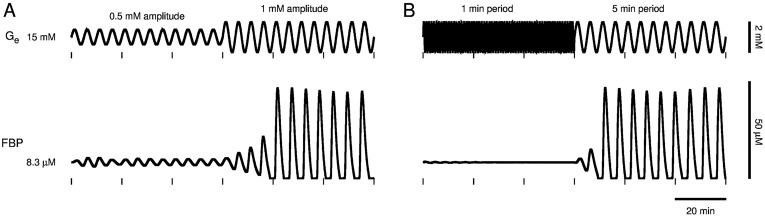
Model glycolytic oscillator recruitment. A sinusoidal external glucose signal with baseline *G*_*e*_ = 15 mM, greater than the Hopf bifurcation at *G*_mid_ ≈ 14.5 mM, must have sufficiently large amplitude and period to activate the DOM glycolytic oscillator. (A) External glucose oscillations started with 0.5 mM amplitude and 5 min period (*t* < 60 min) trigger large-amplitude FBP oscillations only after the amplitude of glucose oscillations is increased to cross below *G*_mid_ ≈ 14.5 mM (*t* > 60 min). (B) Although glucose oscillations started with 1 mM amplitude and 1 min period (*t* < 60 min) cross below *G*_mid_, they trigger large-amplitude FBP oscillations only after the period of glucose oscillations is increased to 5 min (*t* > 60 min).

To demonstrate the dependence on amplitude, we begin oscillating *G*_*e*_ with 0.5 mM amplitude and 5 min period from the stable steady state at *G*_*e*_ = 15 mM above the Hopf bifurcation at *G*_mid_ ≈ 14.5 mM (Fig 5A, top, *t* < 60 min). This results in small FBP oscillations of the same period (Fig 5A, bottom, *t* < 60 min), indicating the glycolytic oscillator is not activated. At 60 min, we increase the amplitude of the glucose oscillations to 1 mM so that now the glucose level crosses below *G*_mid_ (Fig 5A, top, *t* > 60 min). This produces oscillations in FBP that are much larger (Fig 5A, bottom, *t* > 60 min) indicating the glycolytic oscillator is activated. Thus, if the glucose level is varied sinusoidally so that it crosses below *G*_mid_, then the glycolytic oscillator can be activated.

To demonstrate the dependence on period, we begin oscillating *G*_*e*_ with 1 min period and 1 mM amplitude again from the stable steady state at *G*_*e*_ = 15 mM above *G*_mid_ ≈ 14.5 mM (Fig 5B, top, *t* < 60 min). Although the glucose level crosses below *G*_mid_, the glycolytic oscillator is not activated. At 60 min, we increase the period of the glucose oscillations to 5 min, which activates the glycolytic oscillator. Thus, even if the glucose level is varied sinusoidally so that it crosses below *G*_mid_, it must be varied with sufficiently large period to activate the glycolytic oscillator.

We address the phenomena underlying these observations in the Discussion.

### Sinusoidal variation in glucose can initiate slow Ca^2+^ oscillations in the DOM

**Fig 6:**
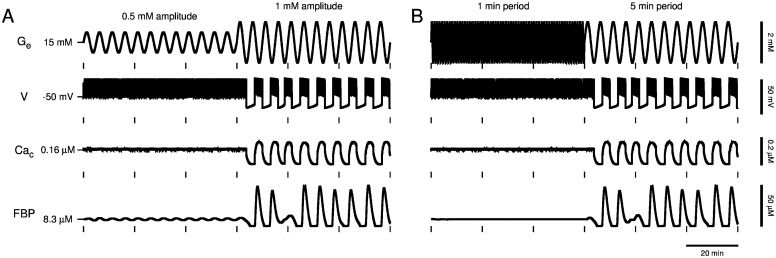
Model islet slow oscillator recruitment. A sinusoidal glucose stimulus with baseline *G*_*e*_ = 15 mM greater than the Hopf bifurcation at *G*_mid_ ≈ 14.5 mM with sufficiently large amplitude and period activates the glycolytic oscillator to recover slow *Ca*_*c*_ oscillations in the DOM. (A) Glucose oscillations started with 0.5 mM amplitude and 5 min period (*t* < 60 min) trigger slow *Ca*_*c*_ oscillations only after the amplitude of glucose oscillations is increased (*t* > 60 min) to activate the glycolytic oscillator. (B) Glucose oscillations started with 1 min period and 1 mM amplitude (*t* < 60 min) trigger slow *Ca*_*c*_ oscillations only after the period of glucose oscillations is increased to 5 min (*t* > 60 min) to activate the glycolytic oscillator.

Next, using the full DOM rather than just the glycolytic subsystem, we simulate the same glucose protocols as in Fig 5 to examine the effect on cytosolic Ca^2+^, *Ca*_*c*_, oscillations (Fig 6). We begin oscillating *G*_*e*_ from the steady state at *G*_*e*_ = 15 mM, above the Hopf bifurcation at *G*_mid_ ≈ 14.5 mM (Fig 6, *t* < 60 min). The glycolytic oscillator is not activated and no slow *Ca*_*c*_ oscillations are produced when glucose oscillations are started with 0.5 mM amplitude and 5 min period (Fig 6A, *t* < 60 min) or with 1 min period and 1 mM amplitude (Fig 6B, *t* < 60 min). Instead, *Ca*_*c*_ is elevated by a continuous spike train of action potentials carried by K(V) and Ca(V) currents. The glycolytic oscillator activates when the amplitude of the glucose oscillations started with 0.5 mM amplitude is increased to 1 mM (Fig 6A, *t* > 60 min) or the period of the glucose oscillations started with 1 min period is increased to 5 min (Fig 6B, *t* > 60 min). When the glycolytic oscillator activates, glycolytic oscillations lead to oscillations in ATP level that, by modulating K(ATP) channels, recover bursting electrical activity and slow *Ca*_*c*_ oscillations (Fig 6, *t* > 60 min). Thus, activation of the glycolytic oscillator underlies the recovery of slow *Ca*_*c*_ oscillations in the DOM, and an oscillatory glucose signal that has sufficiently large amplitude and period can activate the glycolytic oscillator and recover slow *Ca*_*c*_ oscillations.

### Sinusoidal variation in glucose can initiate slow Ca^2+^ oscillations in islets

We tested the model prediction that slow Ca^2+^ oscillations lost in high glucose could be recovered by delivering a sinusoidal glucose signal with sufficiently large amplitude and period. This is performed using our microfluidic platform described in Methods.

**Fig 7:**
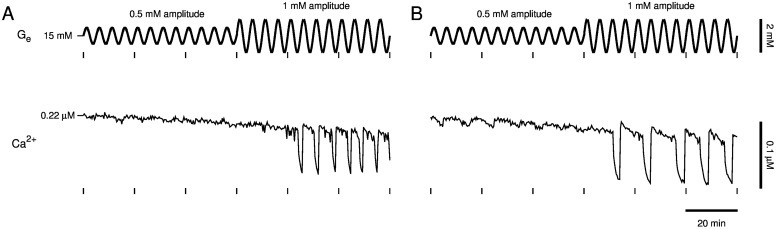
Islet slow oscillator recruitment and dependence on sinusoidal glucose signal amplitude. As predicted by the DOM, an imposed sinusoidal glucose signal must have sufficiently large amplitude to trigger slow Ca^2+^ oscillations in islets. Glucose oscillations started with 0.5 mM amplitude and 5 min period about a 15 mM baseline concentration (*t* < 60 min) triggered slow Ca^2+^ oscillations only after the amplitude of the glucose oscillations was increased to 1 mM (*t* > 60 min). (A) Slow Ca^2+^ oscillations were triggered with an approximately 20 min delay, and (B) in another islet slow Ca^2+^ oscillations were triggered with a period greater than that of the glucose signal, indicating that an endogenous oscillator was activated.

First, we tested the predicted dependence on amplitude (Fig 7). After slow Ca^2+^ oscillations were lost by increasing glucose to 15 mM, we began oscillating glucose with 0.5 mM amplitude and 5 min period for approximately 2 hr. The last hour for two islets is depicted in Fig 7 (top, *t* < 60 min). This did not initiate slow, large-amplitude Ca^2+^ oscillations although small-amplitude oscillations are apparent (Fig 7, bottom, *t* < 60 min). Next, we increased the amplitude of the glucose oscillations to 1 mM (Fig 7, top, *t* > 60 min). In one islet (Fig 7A), this triggered slow Ca^2+^ oscillations with large amplitude after an approximately 20 min delay (Fig 7, bottom, *t* > 60 min). In another islet (Fig 7B), this triggered slow large-amplitude Ca^2+^ oscillations with a period approximately twice that of the imposed glucose signal (Fig 7B, *t* > 60 min). Delayed initiation of Ca^2+^ oscillations and a difference between *G*_*e*_ and Ca^2+^ oscillation periods are evidence that the glucose signal activated an endogenous oscillator with an intrinsic stability and period and did not merely force the islet back and forth between silent and active phases.

**Fig 8:**
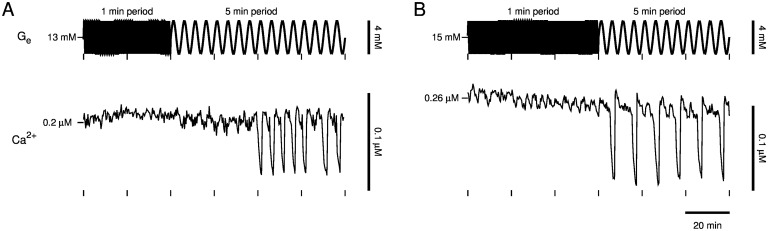
Islet slow oscillator recruitment and dependence on sinusoidal glucose signal period. As predicted by the model, an imposed sinusoidal glucose signal must have sufficiently large period to trigger slow Ca^2+^ oscillations. Glucose oscillations started with 1 min period and 2 mM amplitude about a high baseline concentration (*t* < 60 min) triggered slow Ca^2+^ oscillations only after the period of the glucose oscillations was increased to 5 min (*t* > 60 min). Slow Ca^2+^ oscillations were triggered with (A) an approximately 40 min delay and (B) a period greater than that of the glucose signal in another islet, both indicating an endogenous islet slow oscillator was activated.

Next, we tested the predicted dependence on period. After slow Ca^2+^ oscillations were lost by increasing glucose to a high concentration, we began oscillating glucose with 1 min period and 2 mM amplitude for 1 hr. The last 40 min for one islet are depicted in Fig 8A (top, *t* < 40 min) and the full hour for another islet is depicted in Fig 8B (top, *t* < 60 min). An amplitude of 2 mM, larger than in the simulations depicted in Fig 6, was used in an attempt to trigger slow Ca^2+^ oscillations despite the high-frequency glucose input. However, slow, large-amplitude Ca^2+^ oscillations were triggered only after we increased the period of glucose oscillations to 5 min (Fig 8A, *t* > 40 min and Fig 8B, *t* > 60 min). In one islet, slow Ca^2+^ oscillations were triggered after approximately a 40 min delay (Fig 8A, *t* > 40 min), and in another islet, slow Ca^2+^ oscillations were triggered with a period nearly twice that of the glucose signal (Fig 8B, *t* > 60 min). This is further evidence that the imposed glucose signal activated an endogenous oscillator.

**Fig 9:**
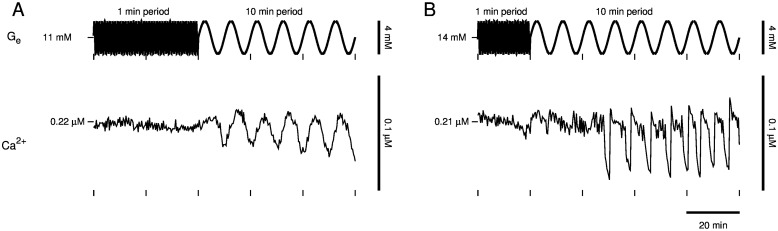
Islet slow oscillator recruitment by sinusoidal glucose signals with 10 min period. Imposed glucose oscillations with a period as slow as 10 min can activate the slow islet oscillator. (A) Slow Ca^2+^ oscillations were entrained by a sinusoidal glucose signal with 10 min period. (B) Slow Ca^2+^ oscillations were triggered with a period approximately half that of the imposed glucose signal in another islet.

What if the imposed glucose signal is even slower? The model predicts that slow Ca^2+^ oscillations could still be recovered. We tested this prediction (Fig 9). After slow Ca^2+^ oscillations were lost by increasing glucose to a high concentration, oscillating glucose with 1 min period and 2 mM amplitude for 1 hr did not trigger slow Ca^2+^ oscillations in two islets (Fig 9A, *t* < 40 min and Fig 9B, *t* < 20 min), yet increasing the period of the glucose oscillations to 10 min triggered slow Ca^2+^ oscillations (Fig 9A, *t* > 40 min and Fig 9B, *t* > 20 min). In one islet (Fig 9A), the slow oscillator was entrained to the glucose signal, evident from synchronization of the Ca^2+^ peaks and glucose nadirs (Fig 9A, *t* > 40 min). In another islet (Fig 9B), Ca^2+^ oscillations emerged with a period approximately half that of the glucose signal (Fig 9B, *t* > 20 min). Thus, imposed glucose oscillations with a period as slow as 10 min can activate the islet slow oscillator.

**Fig 10:**
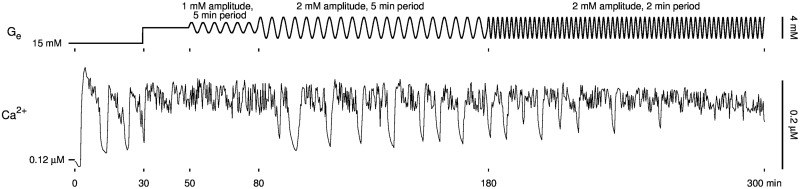
Islet slow oscillator recruitment and dependence on sinusoidal glucose signal amplitude and period. After slow Ca^2+^ oscillations were lost by increasing external glucose concentration to 18 mM at *t* = 30 min, they were recovered at *t* = 80 min by oscillating glucose with sufficiently large amplitude and lost again after *t* = 180 min by decreasing the period of glucose oscillations.

In Fig 10, we depict an experiment that exhibited most of the islet slow oscillator properties mentioned above in a single time course. We started by fixing external glucose at 15 mM (Fig 10, top, *t* < 30 min) which triggered slow Ca^2+^ oscillations (Fig 10, bottom, *t* < 30 min). We then stepped glucose up to 18 mM (Fig 10, top, 30 < *t* < 50 min), which terminated slow Ca^2+^ oscillations (Fig 10, bottom, 30 < *t* < 50 min), indicating that the islet slow oscillator’s predicted *G*_mid_ value is less than 18 mM. We then began oscillating glucose with 1 mM amplitude and 5 min period (Fig 10, top, 50 < *t* < 80 min), which did not recover slow Ca^2+^ oscillations (Fig 10, bottom, 50 < *t* < 80 min), indicating further that *G*_mid_ is less than 17 mM, the minimum of the glucose oscillations during 50 < *t* < 80 min. When we increased the amplitude of the glucose oscillations to 2 mM, slow Ca^2+^ oscillations were recovered with a period approximately three times that of the glucose signal (Fig 10, 80 < *t* < 180 min), but similar to the period of Ca^2+^ oscillations during *t* < 30 min. Finally, when we decreased the period of glucose oscillations to 2 min, slow Ca^2+^ oscillations were eventually lost (Fig 10, *t* > 180 min).

**Fig 11:**
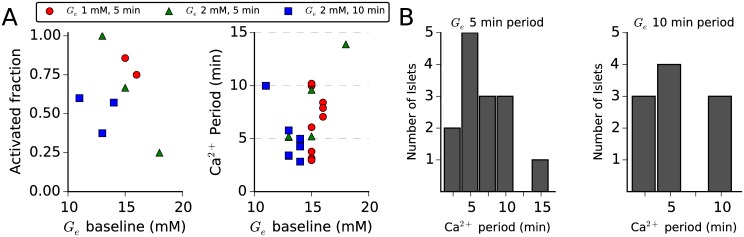
Summary of islet slow oscillator recruitment by a sinusoidal glucose signal. (A) The fraction of islets in which slow Ca^2+^ oscillations were activated and the period of activated Ca^2+^ oscillations versus *G*_*e*_ waveform baseline for the three combinations of *G*_*e*_ amplitude and period considered (1 mM/5 min, 2 mM/5 min, and 2 mM/10 min). For each amplitude and period combination, the activated fraction is generally higher for lower *G*_*e*_ waveform baselines, consistent with the existence of *G*_mid_. Ca^2+^ oscillation periods varied from 2 min to 14 min (mean: 6.56 min, standard deviation: 2.99 min). (B) Histograms of the period of activated Ca^2+^ oscillations by *G*_*e*_ waveforms with 5 min (left) or 10 min (right) periods. Regardless of the glucose waveform period, the majority of activated Ca^2+^ oscillations had a period closer to 5 min than to a multiple of 5 min.

In summary, slow Ca^2+^ oscillations were activated by glucose oscillations in 24 of 40 islets: in 9 of 11 islets by a *G*_*e*_ waveform with 1 mM amplitude and 5 min period, in 5 of 9 islets by a *G*_*e*_ waveform with 2 mM amplitude and 5 min period, and in 10 of 20 islets by a *G*_*e*_ waveform with 2 mM amplitude and 10 min period. Slow Ca^2+^ oscillations were activated in 10 of 15 islets in which the amplitude of the *G*_*e*_ waveform was stepped up (as in Fig 7), and in 14 of 25 islets in which the period of the *G*_*e*_ waveform was stepped up (as in Figs 8 and 9). The period of activated Ca^2+^ oscillations was closer to 5 min than to a multiple of 5 min in 15 of the 24 activated islets, regardless of the *G*_*e*_ waveform period: 8 of 14 islets by a *G*_*e*_ waveform with 5 min period and 7 of 10 islets by a *G*_*e*_ waveform with 10 min period. The effect of stepping the *G*_*e*_ waveform period down to 2 min after stepping the *G*_*e*_ waveform amplitude up to recover slow Ca^2+^ oscillations (as in Fig 10) was studied in 10 islets. Of those 10 islets, slow Ca^2+^ oscillations were initially recovered in 5 islets, and of those 5 islets, slow oscillations were subsequently terminated by stepping down the *G*_*e*_ waveform period in 3 islets. In Fig 11A, we plot the fraction of activated islets and the period of activated Ca^2+^ oscillations versus *G*_*e*_ waveform baseline, and in Fig 11B, we plot histograms of the period of activated Ca^2+^ oscillations by *G*_*e*_ waveforms with 5 min or 10 min period. The period of Ca^2+^ oscillations more similar to 10 min than to another multiple of 5 min activated by a *G*_*e*_ waveform with 5 min period was not significantly different than 10 min (5 islets, two-tailed t-test, *p* = 0.16). Likewise, The period of Ca^2+^ oscillations more similar to 5 min than to another multiple of 5 min activated by a *G*_*e*_ waveform with 10 min period was not significantly different than 5 min (7 islets, two-tailed t-test, *p* = 0.07).

### Slow-oscillating islets can recruit fast-oscillating islets through negative feedback

**Fig 12:**
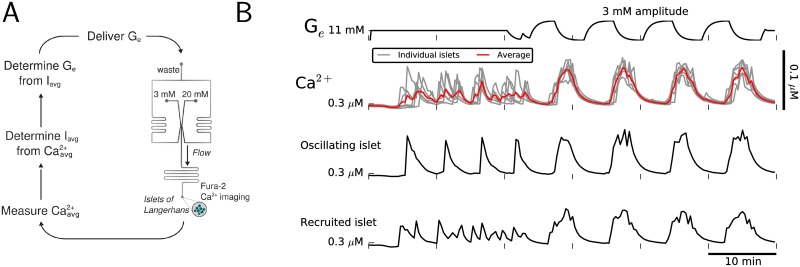
Recruitment of fast-oscillating islets by slow-oscillating islets through negative feedback. (A) Schematic of a closed loop system in which glucose is delivered to islets in the chamber of a microfluidic device and a model liver controls glucose delivery. Average insulin, *I*_*avg*_, modeled as a function of average intracellular Ca^2+^ *Ca*_*avg*_, is used in a differential equation for *G*_*e*_ to continuously update the concentration of delivered glucose. (B) A population of 6 islets is initially delivered a constant 11 mM glucose signal (*t* < 20 min), then interacts in the closed loop (*t* > 20 min). When the loop was closed, 4 slow-oscillating islets synchronized and recruited 2 fast-oscillating islets to amplify the average Ca^2+^ oscillation.

As mentioned above, a constant elevated glucose stimulus may produce pulsatile insulin secretion from only a sub-population of the pancreatic islets. That sub-population of oscillating islets may, through insulin-dependent hepatic control of blood glucose, establish blood glucose oscillations that recruit non-oscillating islets by the mechanism examined in the last section. We tested this hypothesis using a closed loop system, previously described in [20], consisting of the microfluidic device described in Methods and a model liver that controls glucose delivery to the islets in the device chamber (Fig 12A). When the loop is closed, the insulin output from the islet population is modeled as a function of average intraceulluar Ca^2+^ concentration:

$$\begin{array}{cc} I_{avg} & =\max(0,I_{slope}\left(Ca_{avg}-Ca_{thr}\right)), \end{array}$$

and the level of *G*_*e*_ delivered to the islets is continuously updated according to:

$$\begin{array}{cc} \frac{dG_{e}}{dt} & =\frac{G_{\infty }\left(I_{avg}\right)-G_{e}}{\tau_{G}}\\ G_{\infty }\left(I_{avg}\right) & =G_{min}+\frac{G_{max}-G_{min}}{1+\exp[-\left(I_{avg}-I_{1/2}\right)/S_{G}]}. \end{array}$$

Notice, since the dose-response curve *G*_∞_ is a decreasing function of *I*_*avg*_, that this supplies negative feedback to insulin secretion. Parameter values for these equations are listed in S1 Model Parameter Values.

Fig 12B depicts the *G*_*e*_ and Ca^2+^ time courses for 6 islets initially stimulated by a constant 11 mM glucose signal (*t* < 20 min), then interacting in the closed loop system (*t* > 20 min). The initial 11 mM glucose signal triggered slow Ca^2+^ oscillations in 4 islets and fast Ca^2+^ oscillations in 2 islets, indicating *G*_mid_ is greater than 11 mM in the 4 slow-oscillating islets but less than 11 mM in both fast-oscillating islets. Before the loop was closed, Ca^2+^ oscillations were not synchronized among the population, resulting in only small-amplitude oscillations in the average Ca^2+^ time course. When the loop was closed, *G*_*e*_ oscillations with ≈ 3 mM amplitude emerged and recruited the fast-oscillating islets, indicating *G*_mid_ is greater than 8 mM in both of the fast-oscillating islets, the minimum concentration of *G*_*e*_ oscillations. When the loop was closed, Ca^2+^ oscillations were synchronized among the population, resulting in large-amplitude slow oscillations in the average Ca^2+^ time course. Thus, a sub-population of islets that exhibit slow oscillations in response to an elevated glucose concentration can recruit fast-oscillating islets through negative feedback to amplify the average Ca^2+^ oscillation. We also found with the model that when 20 out of 25 islets in a population, heterogeneous in their *G*_mid_ values, initially exhibit slow oscillations in response to a constant 15 mM glucose signal, the other 5 islets could be recruited and the average Ca^2+^ oscillation amplified when the loop was closed.

## Discussion

We have demonstrated that slow Ca^2+^ oscillations in pancreatic islets lost by fixing glucose at an elevated concentration can be recovered by delivering a sinusoidal glucose signal with sufficiently large amplitude and period. This was predicted by our model of islet *β*-cells, which postulates that the sinusoidal glucose signal triggers an endogenous glycolytic oscillator (Fig 5) and that triggering of the glycolytic oscillator underlies recovery of slow Ca^2+^ oscillations (Fig 6). We obtained experimental support for these predictions using a microfluidics platform (Figs 7–11). Importantly, Ca^2+^ oscillations were often triggered after a delay or with a period significantly different than that of a sinusoidal glucose signal, consistent with the activation of an endogenous islet oscillator, rather than a situation in which the islet is forced back and forth between inactive and active states. The data therefore provide further support for the key hypothesis of the Dual Oscillator Model: glycolytic oscillations endogenous to *β*-cells drive slow Ca^2+^ oscillations and pulsatile insulin secretion. The data also provide evidence for the existence of a subcritical Hopf bifurcation at the lower end (*G*_*e*_ = *G*_mid_) of a glucose range of bistability (*G*_mid_ < *G*_*e*_ < *G*_up_), in which oscillations may or may not occur. Furthermore, we have demonstrated, by coupling a model liver to microfluidic platform in a closed loop system, that a sub-population of islets, which respond to an elevated glucose stimulus with slow Ca^2+^ oscillations, can recruit the other islets to become slow oscillators and thereby amplify the population average Ca^2+^ oscillation (Fig 12).

Finding that islet slow oscillators were heterogeneous (Fig 1) was not surprising. The values of *G*_low_, *G*_mid_, and *G*_up_ for the glycolytic oscillator depend on reaction rates of the glycolytic enzymes in *β*-cells, and specifically on the kinetics of allosteric activation of PFK1 by its product, FBP. We expect that islets are subject to varying genetic and environmental factors by virtue of being heterogeneous in other respects, such as size and cell composition, and being dispersed throughout the pancreas. This would cause islet-to-islet variation in glycolytic enzymes’ catalytic properties.

**Fig 13:**
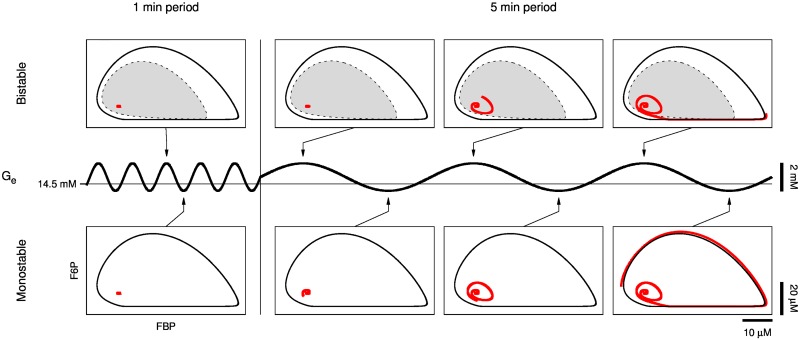
Phase plane analysis of glycolytic oscillator recruitment and dependence on sinusoidal glucose signal period. The glycolytic subsystem transitions between bistable (top) and monostable (bottom) as *G*_*e*_ oscillations cross the Hopf bifurcation at *G*_mid_ ≈ 14.5 mM. When bistable, a stable equilibrium is surrounded by an unstable limit cycle (dashed curve, top), which is surrounded by a stable limit cycle (solid black curve, top and bottom); when monostable, an unstable equilibrium is surrounded by a stable limit cycle. The state of the glycolytic subsystem, i.e. the phase point, trajectory (red curve) is trapped by rapid alternation between disappearance and re-emergence of the stable equilibrium’s domain of attraction (shaded gray) when *G*_*e*_ oscillates with 1 min period (left) but not when when *G*_*e*_ oscillates with 5 min period (right) (see S1 Video for an animated version of this figure).

The prediction that a sinusoidal glucose signal must have sufficiently large amplitude to trigger slow oscillations is simple to explain in terms of the glycolytic oscillator. Starting from a steady state with *G*_*e*_ > *G*_mid_, *G*_*e*_ oscillations with amplitude large enough to cross below *G*_mid_ lead the state of the glycolytic subsystem to the only stable structure that exists for *G*_low_ < *G*_*e*_ < *G*_mid_: a limit cycle. But why must the sinusoidal glucose signal have sufficiently large period to trigger slow oscillations (Figs 5B and 6B)? The answer in terms of the glycolytic oscillator is somewhat subtle and relies on the transition between monostable (Fig 4C, top) and bistable (Fig 4C, bottom) glucose ranges. In Fig 13, we depict the state of the glycolytic subsystem, i.e. the phase point, trajectory (red curve in Fig 13) in the F6P vs. FBP phase plane as *G*_*e*_ oscillations transition from 1 min period (Fig 13, left) to 5 min period (Fig 13, right) as in Fig 5B, 55 < *t* < 75 min (see S1 Video for an animated version of Fig 13). The system alternates between bistable when *G*_*e*_ > *G*_mid_ ≈ 14.5 mM (Fig 13, top) and monostable when *G*_*e*_ < *G*_mid_ (Fig 13, bottom). When bistable, the system has a stable equilibrium surrounded by an unstable limit cycle (dashed curve in Fig 13) which is itself surrounded by a stable limit cycle (solid black curve in Fig 13). The unstable limit cycle is the separatrix between the domain of attraction (shaded gray in Fig 13, top) of the stable equilibrium and the domain of attraction (white in Fig 13 top and bottom) of the stable limit cycle.

Beginning from a steady state with *G*_*e*_ > *G*_mid_ (Fig 13, top left), the phase point is close and attracted to the stable equilibrium point. As *G*_*e*_ drops below *G*_mid_, the equilibrium becomes unstable and it’s domain of attraction vanishes (as the unstable limit cycle and equilibrium point coalesce), so the phase point momentarily remains close to the equilibrium point yet it is attracted to the stable limit cycle (Fig 13, bottom left). The time spent below *G*_mid_ during a cycle of *G*_*e*_ oscillations with 1 min period is so brief that before the phase point can approach the stable limit cycle, the stable equilibrium’s domain of attraction re-emerges and traps the phase point as *G*_*e*_ increases past *G*_mid_. That is, the speed of the phase point trajectory is slow compared to the speed at which the stable equilibrium’s domain of attraction vanishes and re-emerges by *G*_*e*_ oscillations with 1 min period, and therefore the phase point cannot escape the stable equilibrium’s domain of attraction. The situation changes when the period of *G*_*e*_ oscillations increases to 5 min (Fig 13, right). Now, when *G*_*e*_ drops below *G*_mid_ the phase point travels a significant distance from the unstable equilibrium towards the stable limit cycle (Fig 13, bottom center). As *G*_*e*_ increases past *G*_mid_, the stable equilibrium’s domain of attraction re-emerges and transiently traps the phase point (Fig 13, top center). However, after a few cycles, the phase point escapes the stable equilibrium’s domain of attraction and approaches the stable limit cycle (Fig 13, top right). Afterwards, the phase point continues to follow the stable limit cycle (Fig 13, bottom right), producing the glycolytic oscillations that result in slow Ca^2+^ oscillations in our model and, presumably, in islets.

The ability to trigger the glycolytic oscillator with glucose oscillations requires that (1) the baseline glucose level is above *G*_mid_, (2) the oscillation amplitude is large enough to cross below *G*_mid_, and (3) the oscillation period is large enough that the phase point can escape the stable equilibrium’s domain of attraction. Satisfying these three conditions simultaneously can be difficult, and this likely accounts for the imperfect rate of successfully recovering slow Ca^2+^ oscillations in our experiments (10 out of 15 for the protocol of Fig 7 and 14 out of 25 for the protocols of Figs 8 and 9). On the other hand, requirement (2) may not strictly be necessary since inherent noise may perturb the phase point out of the stable equilibrium’s domain of attraction when *G*_*e*_ is in the bistable range (*G*_mid_ < *G*_*e*_ < *G*_up_) yet never crosses below *G*_mid_. Another factor that would contribute to the imperfect success rate is that the extent of bistability will likely exhibit islet-to-islet heterogeneity, and may be quite small in some islets. If the bistable region is too small, then one would not expect a glucose waveform to trigger sustained slow oscillations.

In our model, the slow Ca^2+^ oscillations triggered by an oscillatory *G*_*e*_ signal are stable and therefore persist indefinitely, in the absence of noise or other external stimuli, if *G*_*e*_ oscillations are removed and *G*_*e*_ is held at the signal’s baseline concentration. However, we found that when noise is incorporated into the maximal rate of glucose uptake (*V*_GLUT_) and the baseline *G*_*e*_ concentration is in the bistable range (*G*_mid_ < *G*_*e*_ < *G*_up_), glycolytic oscillations are terminated by noise perturbing the state of the glycolytic subsystem into it’s stable steady state’s domain of attraction. We tested whether Ca^2+^ oscillations persist when *G*_*e*_ oscillations are removed in 15 islets, of which slow Ca^2+^ oscillations were recovered in 11 islets by a *G*_*e*_ waveform with 2 mM amplitude: 4 of 6 islets by a *G*_*e*_ waveform with baseline 13 mM, 3 of 4 islets by a *G*_*e*_ waveform with 15 mM baseline, and 4 of 5 islets by a *G*_*e*_ waveform with 16 mM baseline. Of those 11 islets, slow Ca^2+^ oscillations did not persist for longer than one period after *G*_*e*_ oscillations were removed.

As mentioned above, we observed that stepping the period of a *G*_*e*_ waveform that recovered Ca^2+^ oscillations down to 2 min terminated Ca^2+^ oscillations (as in Fig 10) in 3 of 5 islets. Of those 5 islets, the period of recovered Ca^2+^ oscillations varied considerably in 3 islets before stepping down the *G*_*e*_ waveform period, yet Ca^2+^ oscillations were terminated in only 2 of those 3 islets. Likewise, the period of recovered Ca^2+^ oscillations was nearly constant in 2 islets before stepping down the *G*_*e*_ waveform period, yet Ca^2+^ oscillations were terminated in 1 of those 2 islets. Out of the 24 islets in which Ca^2+^ oscillations were recovered, oscillations persisted for 100 min or longer in 5 islets, all with varying period. Therefore, variation in Ca^2+^ period (as in Fig 10, 80 < *t* < 180 min) does not seem to indicate loss of Ca^2+^ oscillations unless accompanied by change in the *G*_*e*_ waveform. In the model, we found that if Ca^2+^ oscillations were recovered by an oscillatory *G*_*e*_ signal with baseline in the bistable range, stepping the period or amplitude down may or may not terminate the recovered Ca^2+^ oscillations. Whether or not the transition terminates the recovered oscillations depends on the value of *G*_mid_.

The manner in which we manipulated the *G*_*e*_ waveform amplitude and period to activate Ca^2+^ oscillations supports bistability in the islet slow oscillator as described above. Activating Ca^2+^ oscillations by stepping down *G*_*e*_ waveform baseline while keeping oscillation amplitude and period constant would demonstrate the presence of an upper oscillation threshold, but it would not uniquely support the existence of bistability in the islet slow oscillator. Indeed, if the islet slow oscillator exhibited stable oscillations only for intermediate glucose levels (*G*_low_ < *G*_*e*_ < *G*_up_) and exhibited stable steady states only for low (*G*_*e*_ < *G*_low_) and high (*G*_*e*_ > *G*_up_) glucose levels, then Ca^2+^ oscillations could be activated by stepping the *G*_*e*_ waveform baseline from a high to intermediate level. Nonetheless, activating Ca^2+^ oscillations by stepping down the *G*_*e*_ waveform baseline is still consistent with our model, and we observed that stepping down the *G*_*e*_ waveform baseline activated Ca^2+^ oscillations in 18 out of 27 islets.

Are there alternate explanations for the activation of Ca^2+^ oscillations by *G*_*e*_ oscillations? The most obvious alternative, which we refer to as the *push-pull mechanism*, is that the glucose waveform moves the system back and forth between periods when the cell spikes continuously and periods when it is silent, which does not require an endogenous oscillator at all. If this was the case, however, the period of the Ca^2+^ oscillations should equal the period of the glucose waveform. Yet, our data show the Ca^2+^ oscillations that result from the glucose waveform can be faster or slower than the waveform (Fig 11B). This is inconsistent with the push-pull mechanism. A second alternate explanation is that islets have an endogenous oscillator, but it is not the proposed glycolytic oscillator. This could be true as long as the oscillator has a similar bifurcation structure. However, we are unaware of what this alternate endogenous oscillator could be, while there is now ample direct evidence for a glycolytic oscillator in islet *β*-cells [26–28].

The findings presented here have potential physiological implications. If glucose levels sensed by islets are oscillatory, and of sufficiently large amplitude and period, then some islets that would not secrete insulin in a pulsatile manner could be recruited to do so, and this would be beneficial to glucose homeostasis. Few glucose measurements have been made with the temporal resolution needed to detect oscillations with period 5 min or less. In one study in humans, blood sampling done every minute revealed oscillations in glucose of ≈ 0.25 mM from nadir to peak [29]. These oscillations are smaller than those imposed in the current study, but they could be amplified by the islets themselves, which take up and metabolize glucose. Indeed, *in vitro* studies of mouse islets made with glucose-sensing electrodes showed fast (≈ 15 sec) or slow (≈ 3 min) intra-islet glucose oscillations even at a static bath glucose level. These oscillations ranged from 0.2 mM in the case of fast oscillations to 3 mM in the case of slow oscillations, from nadir to peak [30]. The amplitude of these latter oscillations is similar to that of the glucose oscillations imposed in the current study.

## Supporting Information

S1 Model Equations Equations for the Dual Oscillator Model. (PDF)

S1 Model Parameter Values Parameter values of the Dual Oscillator Model used for all simulations and parameter values for the closed loop system used in Fig 12. (PDF)

S1 Video Phase plane animation of glycolytic oscillator recruitment and dependence on sinusoidal glucose signal period. An animated version of Fig 13. The glycolytic subsystem transitions between bistable and monostable as *G*_*e*_ oscillations cross the Hopf bifurcation at *G*_mid_ ≈ 14.5 mM. When bistable, a stable equilibrium is surrounded by an unstable limit cycle (dashed curve), which is surrounded by a stable limit cycle (solid black curve); when monostable, an unstable equilibrium is surrounded by a stable limit cycle. The state of the glycolytic subsystem, i.e. the phase point, trajectory (red curve) is trapped by rapid alternation between disappearance and re-emergence of the stable equilibrium’s domain of attraction (shaded gray) when *G*_*e*_ oscillates with 1 min period (left) but not when when *G*_*e*_ oscillates with 5 min period (right).
(MP4)
