# Supplementary material for: Glucose Oscillations Can Activate an Endogenous Oscillator in Pancreatic Islets
Source: PLoS Comput Biol. 2016 Oct 27;12(10):e1005143. doi: 10.1371/journal.pcbi.1005143 (PMC5082885; doi:10.1371/journal.pcbi.1005143)
Supplement: S1 Model Equations — (PDF) [file pcbi.1005143.s001.pdf]

Supporting Information

S1 Model Equations

The Dual Oscillator Model equations are as follows.
Glycolysis:

$$dG_i/dt = J_{\mathrm{GLUT}} - J_{\mathrm{GK}}$$
$$d\mathrm{F6P}/dt = (1 + k_{\mathrm{GPI}})^{-1}(J_{\mathrm{GK}} - J_{\mathrm{PFK}})$$
$$d\mathrm{FBP}/dt = (1 + k_{\mathrm{LG}})^{-1}(J_{\mathrm{PFK}} - J_{\mathrm{PDH}})$$
$$J_{\mathrm{GLUT}} = V_{\mathrm{GLUT}}\left(\frac{G_e}{G_e + k_{\mathrm{GLUT}}^{\mathrm{GLC}}} - \frac{G_i}{G_i + k_{\mathrm{GLUT}}^{\mathrm{GLC}}}\right)$$
$$J_{\mathrm{GK}} = V_{\mathrm{GK}}\frac{G_i^{h_{\mathrm{GK}}^{\mathrm{GLC}}}}{G_i^{h_{\mathrm{GK}}^{\mathrm{GLC}}} + k_{\mathrm{GK}}^{h_{\mathrm{GK}}^{\mathrm{GLC}}}}$$
$$J_{\mathrm{PFK}} = V_{\mathrm{PFK}}\frac{(1 - k_{\mathrm{PFK}})w_{1110} + k_{\mathrm{PFK}}\sum_{i,j,l\in\{0,1\}} w_{ij1l}}{\sum_{i,j,k,l\in\{0,1\}} w_{ijkl}}$$
$$w_{ijkl} = \frac{\left(\frac{\mathrm{AMP}}{K_{\mathrm{PFK}}^{\mathrm{AMP}}}\right)^i\left(\frac{\mathrm{FBP}}{K_{\mathrm{PFK}}^{\mathrm{FBP}}}\right)^j\left(\frac{\mathrm{F6P}^2}{K_{\mathrm{PFK}}^{\mathrm{F6P}}}\right)^k\left(\frac{\mathrm{ATP}^2}{K_{\mathrm{PFK}}^{\mathrm{ATP}}}\right)^l}{f_{\mathrm{AMP}}^{ik}f_{\mathrm{FBP}}^{jk}f_{\mathrm{MT}}^{il}f_{\mathrm{BT}}^{jl}f_{\mathrm{ATP}}^{kl}}$$
$$J_{\mathrm{PDH}} = k_{\mathrm{PDH}}\sqrt{\mathrm{FBP}}$$

ATP Production/Hydrolysis:

$$d\mathrm{ATP}/dt = J_{\mathrm{ANT}} - J_{hyd}$$
$$J_{\mathrm{ANT}} = V_{\mathrm{ANT}}\mathrm{ADP}\exp\left[\left(k_{\mathrm{ANT}} + k_{\mathrm{ANT}}^{\mathrm{PDH}}\frac{J_{\mathrm{PDH}}}{J_{\mathrm{PDH}} + V_{\mathrm{ANT}}^{\mathrm{PDH}}}\right)\left(1 - \frac{Ca_c}{k_{\mathrm{ANT}}^{Ca}}\right)\right]$$
$$J_{hyd} = (k_{hyd,bas} + k_{hyd}^{Ca}Ca_c)\mathrm{ATP}$$
$$\mathrm{AMP} = \mathrm{ADP}^2/\mathrm{ATP}$$
$$\mathrm{ATP} + \mathrm{ADP} + \mathrm{AMP} = A_{tot}$$

Membrane Potential and Calcium Concentrations:

$$
\begin{aligned}
dV/dt &= -(I_{Ca(V)} + I_{K(V)} + I_{K(Ca)} + I_{K(\mathrm{ATP})})/C_m \\
dn_{K(V)}/dt &= (n_{K(V),\infty} - n_{K(V)})/\tau_{K(V)} \\
dCa_c/dt &= k^{Ca}_{cyt}(J_{\mathrm{PM}} - J_{\mathrm{ER}}) \\
dCa_{\mathrm{ER}}/dt &= k^{Ca}_{\mathrm{ER}} J_{\mathrm{ER}} \\
I_{i(s)} &= g_{i(s)} n_{i(s)} \cdot (V - V_i), \quad i(s) \in \{Ca(V), K(V), K(Ca), K(\mathrm{ATP})\} \\
n_{i(s),\infty} &= [1 + (k_{i(s)}/r(s))^{h_{i(s)}}]^{-1}, \quad i(s) \in \{Ca(V), K(V), K(Ca)\} \\
n_{i(s)} &= n_{i(s),\infty} \text{ for } i(s) \in \{Ca(V), K(V)\} \\
r(s) &= \begin{cases} e^s & \text{if } s = V \\ s & \text{otherwise} \end{cases} \\
n_{K(\mathrm{ATP})} &= \frac{0.08\left(1 + 2\frac{\mathrm{MgADP}^-}{kdd}\right) + 0.89\left(\frac{\mathrm{MgADP}^-}{kdd}\right)^2}{\left(1 + \frac{\mathrm{MgADP}^-}{kdd}\right)^2 \left(1 + \frac{\mathrm{ADP}^{3-}}{ktd} + \frac{\mathrm{ATP}^{4-}}{ktt}\right)} \\
\mathrm{MgADP}^- &= 0.0165\mathrm{ADP} \\
\mathrm{ADP}^{3-} &= 0.135\mathrm{ADP} \\
\mathrm{ADP}^{4-} &= 0.05\mathrm{ATP} \\
J_{\mathrm{PM}} &= -[I_{Ca(V)}/(2F) + k_{\mathrm{PM}} Ca_c] \\
J_{\mathrm{ER}} &= k_{\mathrm{ER},in} Ca_c - k_{\mathrm{ER},out}(Ca_{\mathrm{ER}} - Ca_c)
\end{aligned}
$$

S1 Text. Model Equations.
Equations for the Dual Oscillator Model.
