# Supplementary material for: Glucose Oscillations Can Activate an Endogenous Oscillator in Pancreatic Islets
Source: PLoS Comput Biol. 2016 Oct 27;12(10):e1005143. doi: 10.1371/journal.pcbi.1005143 (PMC5082885; doi:10.1371/journal.pcbi.1005143)
Supplement: S1 Model Parameter Values — (PDF) [file pcbi.1005143.s002.pdf]

Supporting Information

S1 Model Parameter Values

Parameter	Value	Parameter	Value	Parameter	Value
V_{GLUT}	0.01 M/s	V_{GK}	0.01 M/s	V_{PFK}	0.01 M/s
k_{PDH}	0.001 M/s	$k_{\text{GLUT}}^{\text{GLC}}$	13 mM	k_{GK}	13 mM
$h_{\text{GK}}^{\text{GLC}}$	4	k_{GPI}	3.3	k_{PFK}	0.06
$K_{\text{PFK}}^{\text{AMP}}$	30 μM	$K_{\text{PFK}}^{\text{FBP}}$	1 μM	$K_{\text{PFK}}^{\text{F6P}}$	50 μM
$K_{\text{PFK}}^{\text{ATP}}$	0.1 μM	f_{AMP}	0.02	f_{FBP}	0.2
f_{MT}	20	f_{BT}	20	f_{ATP}	20
k_{LG}	0.3	V_{ANT}	1 s^{-1}	$k_{\text{hyd},bas}$	3.3×10^{-3} s^{-1}
k_{hyd}^{Ca}	0	k_{ANT}	1	$k_{\text{ANT}}^{\text{PDH}}$	2.2
$V_{\text{ANT}}^{\text{PDH}}$	0.05 M/s	k_{ANT}^{Ca}	3.5×10^{-4} μM	A_{tot}	3 mM
V_{Ca}	25 mV	V_K	-75 mV	$g_{Ca(V)}$	1 nS
$g_{K(V)}$	2.7 nS	$g_{K(Ca)}$	50 pS	$g_{K(\text{ATP})}$	25.5 nS
$v_{Ca(V)}$	-20 mV	$v_{K(V)}$	-16 mV	$k_{K(Ca)}$	0.5 μM
$h_{Ca(V)}$	0.08	$h_{K(V)}$	0.2	$h_{K(Ca)}$	2
$\tau_{K(V)}$	20 ms	C_m	5.3 pF	kdd	17 μM
ktd	26 μM	ktt	1 μM	k_{cyt}^{Ca}	0.01
k_{ER}^{Ca}	0.01	$k_{\text{ER},in}$	0.46 s^{-1}	$k_{\text{ER},out}$	0.02 s^{-1}
k_{PM}	0.23 s^{-1}	I_{slope}	1 A.U./nM	Ca_{thr}	0.22 μM
τ_G	50 s	G_{min}	8 mM	G_{max}	14 mM
$I_{1/2}$	30 A.U.	S_G	50 A.U.		

S1 Table. Model parameter values.
Parameter values of the Dual Oscillator Model used for all simulations and parameter values for the closed loop system used in Fig. 12.
